# Contribution of benzodiazepines in dental care of patients with special needs

**DOI:** 10.4317/jced.56149

**Published:** 2019-12-01

**Authors:** Bruna-Lavinas-Sayed Picciani, Bruna-Michalski dos Santos, Geraldo-Oliveira Silva-Júnior, Marcello-Alves Marinho, Eliane-Garritano Papa, Marcelo-Daniel-Brito Faria, Luciana-Freitas Bastos, Cresus-Vinícius-Depes de Gouvêa

**Affiliations:** 1 Postgraduate Program in Dentistry, Universidade Federal Fluminense, Nova Friburgo, RJ, Brazil; 2 Dental Center for Patients with Special Needs, Rir Institute, Rio de Janeiro, RJ, Brazil; 3 Center of Dental Radiology and Care to Patients with Special Needs, Piquet Carneiro Polyclinic, Universidade do Estado do Rio de Janeiro, RJ, Brazil; 4 Department of Diagnosis and Therapeutics, School of Dentistry, Universidade do Estado do Rio de Janeiro, RJ, Brazil; 5 Department of Clinical Dentistry, Universidade Federal Fluminense, Niterói, RJ, Brazil; 6 Department of Preventive and Community Dentistry, School of Dentistry, Universidade do Estado do Rio de Janeiro, RJ, Brazil; 7 Department of Technical Dentistry, Universidade Federal Fluminense, Niterói, RJ, Brazil

## Abstract

**Background:**

Conscious sedation in dental treatment of patients with special needs (PNEs) has the purpose of controlling events such as anxiety and fear, as well as promoting muscle relaxation and mastery of uncoordinated movements. Benzodiazepines (BZDs) are among the most used drugs due to their anxiolytic, hypnotic and sedative properties. The objective of this investigation is to demonstrate a study on the contribution of conscious sedation with BZD in PNEs.

**Material and Methods:**

The study included 40 PNEs, non-collaborators, submitted to conscious oral sedation with Midazolam (0.5 mg/kg) for dental treatment, receiving vital signs monitoring in the pre, trans and postoperative periods.

**Results:**

Male patients were more frequent with 70% of the cases, with a mean age of 18 years. As for medical diagnosis, autism and mental deficiency were the most prevalent. The most performed procedures were restoration (32%) and exodontia (30%). There was a statistically significant reduction in systolic and diastolic blood pressure parameters (*p*<0.05) in the transoperative and postoperative periods when compared to the preoperative period. Conscious sedation with BZDs resulted in 83% positive responses.

**Conclusions:**

The results demonstrate that this technique is safe and effective, and can be used in outpatient care for PNEs. However, the risk/benefit ratio should be correctly evaluated.

** Key words:**Special patients, oral sedation, benzodiazepines, midazolam, dental care.

## Introduction

Dental care for patients with special needs (PNEs) covers a diverse group of people with one or more health conditions, whether chronic or acute, requiring specialized and individualized clinical management (1). One of the great challenges for adequate outpatient care is to make the patient to collaborate, which in many cases does not happen due to fear and anxiety. In addition, the patient may not be able to develop emotional control of these factors or may not have full intellectual development, and their capacity for understanding and cooperation may be affected (1).

The first option is to apply methods of psychological conditioning, however, they may not be sufficient for adequate dental treatment. In these cases, conscious sedation is an effective and safe alternative that allows the patient to become more cooperative, promoting the accomplishment of a less traumatic and more resilient treatment. The main objectives of sedation are reduction of anxiety and fear, as well as mild analgesia and reduction of nausea and salivary flow (2-4).

Among the pharmacological methods of sedation in dentistry, oral benzodiazepines (BZDs) are one of the most widely used alternatives to this degree of sedation (5). They consist of a group of drugs that have proven efficacy and promote safety in clinical use (6). Its action occurs through the interaction of the drug with specific receptors in the central nervous system, promoting sedation, hypnosis, anxiety control, skeletal muscle relaxation, anterograde amnesia, anticonvulsant activity, reduction of salivary flow and vomiting reflex (7,8). Minimal effects may occur in the cardiovascular and respiratory systems, such as mild reduction in blood pressure and heart and respiratory rates (7). The BZDs most used in dentistry are: Diazepam, Lorazepam, Alprazolam, Midazolam and Triazolam, being classified according to the onset of action and the duration of the anxiolytic effect (9,10). Midazolam was the drug of choice for outpatient dental procedures because, compared to other drugs, it allows administration in children, adults and older persons, has a rapid onset of action and presents a short duration of pharmacological effect (11).

Although studies point to oral sedation with BZDs as an effective option in dentistry, there are few studies that have evaluated the effect of this sedation on dental care in patients with special needs.

The objective of this study is to evaluate the contribution of conscious sedation with benzodiazepines at dental care for patients with special needs, demonstrating the indication, safety and efficacy of this technique.

## Material and Methods

The sample of this study consisted of 40 participants, attended at the Odontological Center for Special Patients of the Brazilian Dental Association – RJ (COPE-ABORJ) for dental treatment between 2015 and 2016. The sample included patients with uncontrollable fear or anxiety, neuromuscular disorders, or reduced intellectual capacity, as well as those that did not collaborate or presented conditions that hindered or prevented the initial dental care and that could impair the conduction of the treatment in subsequent consultations.

Instructions for oral sedation were given to inform the patient and/or caregiver/family about the use of benzodiazepine, possible adverse and side effects of the previous and posthumous sedation. For a safe dental treatment, a request for medical opinion on oral sedation was issued for all candidates in order to complement pertinent clinical information as well as medications for daily use and possible care needed during it.

Patients were submitted to oral sedation with compressed Midazolam at doses of 7.5 or 15 mg or with titration based on application of 0.5 mg/kg, not exceeding 20 mg per consultation. Patients who were unfit for and/or who did not respond satisfactorily after oral sedation were referred for hospital-level treatment under general anesthesia. During clinical care, blood pressure, heart rate and oxygen saturation were monitored in the pre, trans and postoperative periods, at intervals of 15 minutes between each; results were obtained through digital sphygmomanometer and pulse oximeter.

The demographic and clinical data collected from patients corresponded to sex, skin color, age, body mass and diagnosis. Due to the difficulty in accurately classifying all participants, the skin color was classified only into white, black and brown.

This study was approved by the Research Ethics Committee of the Hospital Universitário Pedro Ernesto, Universidade do Estado do Rio de Janeiro (HUPE/UERJ) with the number CAAE: 24279314.1.00005259.

All statistical tests used in this study were performed through the statistical program SPSS (Statistical Package for the Social Sciences, version 22.0). The statistical description of the studied variables covered standard deviations, minimum-maximum and median values (age, blood pressure, heart rate and O2 saturation). The Student’s t-test was used to evaluate the differences between continuous quantitative variables, i.e. in the preoperative, intraoperative and postoperative periods, two to two, for analysis of blood pressure, heart rate and O2 saturation alone.

## Results

The male sex prevailed with 28 (70%) patients and the most frequent skin color was white with 28 (70%) cases. The age ranged from 6 to 73 years, with an average of 18 years (SD=14 years). In relation to body mass, the mean was 46 kg (SD=±26 kg), and male patients presented a higher body mass in absolute values, 55 kg (SD=±22 kg), table 1. Of the 40 patients with special non-employee needs, autism prevailed (28%), followed by intellectual disability (15%), and Down syndrome (12%) (Table 1). Among the dental procedures performed, restoration prevailed with 32%, followed by dental extraction with 30% ( Table 1).

**Table 1:**
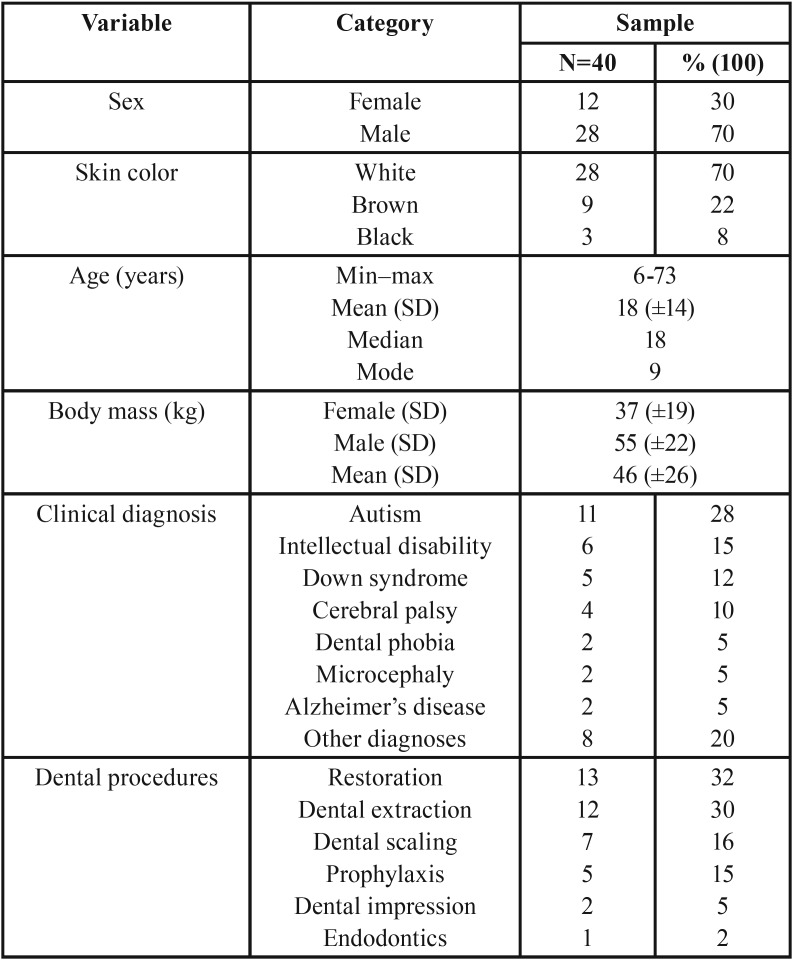
Distribution of sample according to demographic and clinical profile.

In relation to vital signs monitoring, these parameters were reduced to preoperative and trans-operative periods, with a mean decrease of 2 mmHg in systolic blood pressure and 1 mmHg in diastolic blood pressure; and a reduction of 6 bpm in heart rate on average. In no case did the oxygen saturation reach below 96%. There was a statistically significant reduction in systolic and diastolic blood pressure parameters, as well as heart rate, between the pre and postoperative periods, demonstrating the efficacy of BZDs in anxiety control ( Table 2). There was a statistically significant reduction in blood pressure and heart rate (*p*<0.01, Student’s t-test), with maintenance of satisfactory levels of oxygen saturation. In most cases (83%), sedation was shown to be safe and effective, as reported by professionals.

**Table 2:**
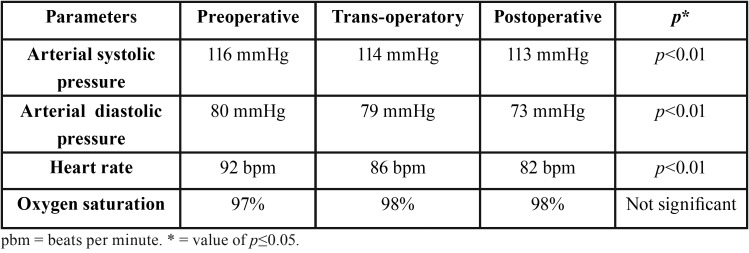
Parameters measured during the pre, trans and postoperative periods.

## Discussion

The use of various pharmacological methods of sedation in dentistry has been increasing with the passage of time. Numerous studies are carried out with the purpose of describing, analyzing and improving the techniques and results in the dental care of patients with diagnoses or clinical conditions that make it difficult for the professional to handle, thus enabling appropriate outpatient treatment with benefits for patients and professionals. This study was developed based on this assumption and focused on specific objectives in order to asses the contribution that oral sedation would have in the dental management of special non-collaborating patients (12).

These studies are needed to describe the profile of patients who require sedation for psychological control of fear and anxiety, the behavior of non-cooperative patients, and the patients’ response to sedation in terms of results of clinical data, such as decreased anxiety/fear or increased cooperation during consultations. To the best of out knowledge, the literature presents no investigation with a comparative methodology similar to our study, even though it is necessary to study the population with clinical focus and show the real contribution of oral sedation for anxiety control and behavior of the non- cooperative patient in dental care.

In this study, we used nasal or venous drugs to control anxiety in dentistry (13-15). The objective was to describe the mechanism of action of the drugs used, not the clinical signs such as blood pressure, oxygen saturation and the type of procedure performed. Another fact that calls our attention is the type of indication or the type of patient who underwent oral sedation, which for us seems to be overwhelmed, inspiring more assistance for pediatric patients without comorbidities and adults with fear and anxiety.

The prevalence of males in this pilot study was higher (70%), as in the study by Moreira *et al.*, with 56% of the sample consisting of males (16); however, other studies reported having prevalence of female patients with 73%, 66% and 64%, respectively (13,17,18). According to our results, it can be assumed that sex is not a determining factor that expresses the need for pharmacological sedation.

The mean age in the studies of Jerath *et al.* and Masuda *et al.* were close, with the first one being 40, with a minimum age of 20 years and a maximum of 85, and the second study being 47 years, varying from 9 to 85 years old (14,19). When compared to the mean of our study (18 years), it can be observed that the means were higher; however, variations of ages were close to the present study, which presented ages from 6 to 78 years.

On the other hand, studies showed much lower mean age results, being 3 years and 4 months (40 months) and 2 years and 3 months (28 months), respectively, which may be justified because they are studies that have addressed the use of sedatives in pediatric patients (16,17).

When analyzing the results with respect to age, initially one can think of two possibilities: the first is that behavior management techniques and pediatric patient iatrosedation have not been enough in conditioning for dental treatment. The second may be related to the increase in the life expectancy of the general population and the need for more complex and invasive procedures, associated with the accumulation of comorbidities in these patients.

In fact, when we compare our results with the reasoning described previously, we believe that some limitations can be pointed out and increased in a more in-depth study and with a larger number of patients.

These limitations are added to future studies that, besides collecting the data described by us, should also add relationships such as the type of medication that the patient uses associated with the type of medication used for sedation, as well as his or her medical diagnosis. These variables can answer why there were cases where sedation had no effect or had complications during the procedure, as paradoxical effect, retrograde amnesia and profound sleepiness, for example.

## Conclusions

In conclusion, benzodiazepine sedation, when well employed, is a safe and effective technique, constituting an option for ambulatory care of patients with special non-cooperative needs. However, thorough knowledge of this technique, its risks and benefits, and the monitoring of vital signs (blood pressure, heart rate and oxygen saturation) of the patient are essential.
